# RURAL–URBAN DIFFERENCES IN POSTSURGICAL SERVICE USE AND OUTCOMES AMONG VETERANS WITH DEMENTIA

**DOI:** 10.1093/geroni/igad104.0571

**Published:** 2023-12-21

**Authors:** Heather Davila, Yubo Gao, Michael Jacobs, Katherine Hadlandsmyth, Carly Duncan, Andrea Strayer, Daniel Hall, Mary Vaughan-Sarrazin

**Affiliations:** Iowa City VA Healthcare System, Iowa City, Iowa, United States; University of Iowa Carver College of Medicine, Iowa City, Iowa, United States; VA Pittsburgh Healthcare System, Pittsburgh, Pennsylvania, United States; Iowa City VA Health Care System, Iowa City, Iowa, United States; VA Pittsburgh Healthcare System, Pittsburgh, Pennsylvania, United States; University of Iowa, Iowa City, Iowa, United States; VA Pittsburgh Healthcare System, Pittsburgh, Pennsylvania, United States; University of Iowa Carver College of Medicine, Iowa City, Iowa, United States

## Abstract

Adults with dementia have worse post-surgical outcomes than those without dementia, including readmission and death. The use of home health or facility-based skilled nursing after discharge may prevent poor outcomes. We know little about rural-urban differences in post-discharge services or outcomes among individuals with dementia. We examined the relationship between post-discharge service use and 30-day readmission or mortality using retrospective data for Veterans Affairs patients aged 65+ with dementia who underwent inpatient surgery between 2013-2019. We used merged VA and Medicare claims to categorize patients as discharged to home without services, home with home health, or skilled nursing/rehabilitation facility [SNF]. We excluded individuals who were transferred to another acute hospital, transferred to hospice, died or were readmitted within 3 days of discharge, or were not enrolled in Medicare. We used multivariable logistic regression to assess associations between rurality, discharge location, and outcomes, while controlling for demographics, frailty, comorbidities, surgical specialty, and surgical complexity. The analytic sample included 4534 Veterans; 48.7% discharged to a SNF, 40.1% to home without services, and 10.4% to home with home health. We did not find rural-urban differences in risk-adjusted readmission or mortality overall. Among those discharged to home (n=2324), receipt of home health was modestly associated with decreased readmission risk overall (OR=0.52; p=0.07), and lower risk of morality among urban Veterans (OR=0.34; p=0.05) but not rural Veterans. Although we did not find rurality disparities in outcomes, further research may identify reasons home health decreases post-surgical mortality among urban but not rural adults with dementia.

